# Assessing Factors Associated With Survival Among Cervical Cancer Patients in Kenya: A Retrospective Follow-up Study

**DOI:** 10.24248/EAHRJ-D-18-00010

**Published:** 2018-11-23

**Authors:** Damar Osok, Simon Karanja, Yeri Kombe, Eliud Njuguna, Jim Todd

**Affiliations:** a School of Public Health, Jomo Kenyatta University of Agriculture and Technology, Nairobi, Kenya; b Centre for Public Health Research, Kenya Medical Research Institute, Nairobi, Kenya; c Cancer Treatment Centre, Kenyatta National Hospital, Nairobi, Kenya; d Department of Population Health, London School of Hygiene and Tropical Medicine, London, UK

## Abstract

**Background:**

Cervical cancer ranks as the fourth most commonly diagnosed cancer and the fourth leading cause of cancer death in women worldwide. In Kenya, cervical cancer is the second most commonly diagnosed cancer after breast cancer and the leading cause of cancer death in women. It is estimated that by the end of 2018, cervical cancer will be responsible for 5,250 (11%) new cases and 3,286 (11.84%) deaths in Kenya.

**Methods:**

We conducted a retrospective follow-up study to estimate the overall survival of women treated for cervical cancer in Kenya. Medical records were reviewed to extract information for generating a quantitative data set, and the chi-square test was used to test for associations between patient outcomes and various sociodemographic and clinical factors. To estimate overall survival after treatment, we used Kaplan–Meier survival analysis, the logrank test, and Cox proportional hazards regression.

**Results:**

A total of 481 patient records were included in this study. From the bivariate analysis, 4 factors demonstrated a statistically significant association with survival: access to care (*P*=.049), stage of disease at diagnosis (*P*<.001), type of treatment received (*P*<.001), and whether or not treatment was initiated and completed (*P*<.001). The overall 5-year survival estimate for women with cervical cancer was 59%. However, 396 (82.3%) women were lost to follow-up; with no deaths observed after the first year, the overall survival estimate is only accurate for the first year.

**Conclusion:**

The high rate of loss to follow-up appears to be characteristic of cancer care in Kenya and highlights the difficulties in conducting survival studies in low-resource settings with low coverage of vital registration and a lack of centralised national administrative systems. Despite the study's limitations, the results support evidence whereby late-stage diagnosis, deficiencies in cancer management, and limited cancer care services, in particular, have been found to contribute to poor patient outcomes in sub-Saharan Africa.

## INTRODUCTION

The Global Cancer Incidence, Mortality, and Prevalence (GLOBOCAN) database indicates that, in 2018, cervical cancer will be responsible for 570,000 new cases and 311,000 deaths globally.^1^ Cervical cancer, therefore, ranks as the fourth most commonly diagnosed cancer and the fourth leading cause of cancer death in women worldwide.^1^ Although cervical cancer incidence and mortality rates have been on the decline in many populations worldwide, evidence suggests that these rates are increasing in sub-Saharan Africa.^2,3^ This is catastrophic considering the highest regional age-standardised incidence rates (ASIR) and mortality rates (ASMR) for cervical cancer are in Africa, with particularly elevated rates observed in southern (ASIR, 43.1; ASMR, 20.0), eastern (ASIR, 40.1; ASMR, 30.0), and western Africa (ASIR, 29.6; ASMR, 23.0).^1^ In Kenya specifically, it is estimated that, in 2018, cervical cancer will be responsible for 5,250 (11%) new cases and 3,286 (11.84%) deaths. This makes cervical cancer the second most commonly diagnosed cancer in Kenya after breast cancer and the leading cause of cancer death in women.^4^

Timely pathology and laboratory services are fundamental for the provision of quality health services for noncommunicable diseases (NCDs).^5^ However, few countries have any or enough staff to adequately support the need. For example, only Botswana and South Africa have at least 1 pathologist for every 500,000 people, while Somalia does not have any active pathologists.^6^ Factors driving poor access to care and low survival in sub-Saharan Africa include: few trained health workers, poor health system infrastructure, and high cost of oncological care in the absence of universal health care.^7^ In Kenya, breast and cervical cancer treatment services in the private sector were found to be 10 times more expensive compared to the public sector.^8^ Without health insurance, even care at a public hospital becomes inaccessible for many.^8^

Cancer is not a rare disease in Africa. However, the overwhelming burden of communicable diseases has restricted investments in appropriate cancer control strategies and management guidelines and has resulted in late-stage diagnosis of cancer with poor outcomes.^9^ For example, analysis of population-based cancer survival data found that 5-year age-standardised relative survival did not exceed 22% for any cancer site in The Gambia or 13% for any cancer site except the breast (46%) in Uganda.^10^ This translates to a high proportion of terminally ill cancer patients; data suggest that at least 88% of cancer deaths in Africa with moderate to severe pain are untreated.^11^ In Kenya, the scaling up of palliative care services faces 3 main challenges: difficulties in forecasting demand for opioid analgesics, administrative bottlenecks that characterise the public-sector procurement process, and a lack of sufficient funding for essential drugs including morphine.^11^

Very little is known about the structure, processes, and outcomes of cancer control activities in sub-Saharan Africa. Development of high-quality health data sources and improved capacity for health services research would promote better understanding of the current situation and identify areas of improvement of oncological health services in low- and middle-income countries (LMICs).^12^ This study aimed to use medical records to estimate overall survival among cervical cancer patients interacting with the Kenyan health system, which operates a predominantly centralised oncological health service.

## METHODS

This retrospective follow-up study was conducted at Kenyatta National Hospital (KNH), a public teaching and referral hospital with a 1500-bed capacity. The hospital's radiotherapy and obstetrics and gynaecology departments make up the largest cervical cancer management centre in Kenya, receiving patients from all over the country. There was no direct contact between the study team and patients. All information and data presented in this article were solely obtained from the review of clinical notes and laboratory reports contained within study participants' medical records.

### Sample Size

No sample size calculation was made for this study. KNH was the only hospital in Kenya offering comprehensive cancer care in 2008. Therefore, we aimed to identify every case of cervical cancer attended to at the hospital. However, a retrospective sample-size calculation indicates that the study's final sample size of 481 patients would have been sufficient to estimate a mortality rate of 20% per year with a precision of 5%.

### Data Collection

A review of medical records belonging to all cervical cancer patients who presented with illness for the first time between January and December 2008 at KNH was undertaken. We obtained medical records from the hospital's inpatient record registry at the Health Information Department (HID) and from the outpatient record registry at the Radiotherapy Clinic (RTC).

Both the HID and RTC operated predominantly manual paper-based record registry systems. In the HID, a registry clerk identified and retrieved hard-copy patient records based on each files' assigned International Classification of Diseases (ICD) 10 code of C53/C53.9 for cervical cancer. The HID does not code files using all the 10 digits of the code. Within the RTC, the staff advised our team where to physically locate files for all patients treated in their clinic in 2008.

We defined cervical cancer cases as patients with histo-logically confirmed cervical cancer, diagnosed via biopsy. Records of patients who met that definition and commenced treatment at KNH between January and December 2008 were included in the study. Patients' records were excluded from the study if the patients were diagnosed with a form of cancer other than cervical cancer, received a histological diagnosis of benign tumour of the cervix or clinical diagnosis of cervical cancer, or if they commenced treatment at KNH prior to January or after December 2008.

We screened approximately 2,367 patient files opened between January and December 2008 to identify those belonging to cervical cancer patients. Through triangulation of patient data and follow-up between the 2 registries of inpatient and outpatient departments, 617 medical records of the initial 2,367 were confirmed as belonging to cervical cancer patients who sought treatment at KNH between January and December 2008. The 617 medical records were thereafter assessed against the study's inclusion and exclusion criteria.

The major limitation with the patient file retrieval process was that unlike the HID, which codes patient files and retains a separate but equally detailed record registry for patients who died during their admission, the RTC does not code their files according to cancer type nor do they keep an organised record or registry for deceased patients. The deceased patient files are stored in cupboards and date back several decades. Consequently, only patients whose files were considered active in the RTC main registry were reviewed. This means that the number of hospital-occurring deaths was likely underestimated and not all cervical cancer patient files were reviewed.

The study was carried out between February and August 2014. We extracted the required patient information from the 481 patient medical records selected for inclusion in the study using a specially designed data entry form in Epi Info 7 (Centers for Disease Control and Prevention, Atlanta, GA, USA). The data collected included information on sociodemographic factors (eg, age, marital status, education, and parity), clinical factors (eg, diagnostic method, stage of disease, and tumor histopathology), and patient outcomes at 5 years (ie, death, alive at 5 years, or lost to follow-up). This ensured uniformity in data extraction and generated a quantitative data set.

### Data Analysis

Statistical analysis was conducted using Stata, version 14.2 (StataCorp LLC, College Station, TX, USA) after manually exporting the data from the Epi Info database. We generated descriptive statistics for both sociodemographic and clinical factors. Using Pearson's chi-square (X^2^) test, associations between patient outcomes and various sociodemo-graphic and clinical factors considered during this study were assessed.

Overall survival was defined as the length of time a patient was alive from the date of diagnosis to 5 years post diagnosis. Survival analysis was restricted to factors showing strong associations with patient outcomes as determined during bivariate analysis. However, age was included as an additional potential confounder, although it did not demonstrate any statistically significant association with patient outcomes for this study population. Initially, we conducted Kaplan–Meier survival analysis to estimate the mean survival time until death and the median survival time (ie, time at which 50% of subjects had died). The logrank test was thereafter applied to compare the survival distribution between groups. This was followed by mortality hazard ratio analyses. Cox proportional hazards regression was used to generate both univariate and multivariate hazard ratios (ie, rates adjusted for potential confounders). We then compared the univariate and multivariate hazard ratios to establish which factors consistently demonstrated an influence over the overall survival rates of cervical cancer patients treated at KNH. A significance level of .05 and, where appropriate, a 95% confidence interval (CI) were used to interpret the analysis results.

### Ethical Approval

Ethical approval for this study was granted by the Kenya Medical Research Institute (KEMRI) Ethical Review Committee (KEMRI/RES/7/3/1 Protocol SSC No. 2486) and the University of Nairobi/Kenyatta National Hospital Ethical Review Committee (KNH-ERC/R&R/546 Protocol No. P404/7/2013).

## RESULTS

We reviewed a total of 617 medical records for inclusion in this study; 481 records qualified for inclusion while 136 were excluded. Of the 136 excluded records, 97 (71.3%) belonged to women who had presented with illness for the first time at KNH earlier than January 2008. In 18 (13.2%) cases, patients were treated for clinically diagnosed cervical cancer. The remaining 21 (15.5%) cases belonged to patients whose records were miscoded during filing and were either ailing from noncancerous illnesses or diagnosed with cancers other than cervical cancer.

### Sociodemographic and Selected Characteristics

The patients had a mean age of 49 (95% CI, 48.26 to 50.57) years and a median age of 48 years (range, 20 to 86 years). The 40-to 49-year age group was the largest, with 147 (30.6%) patient records. Collectively, 262 women of reproductive age (20 to 49 years) accounted for 54.5% of the study population. Of the 481 patients, 194 (40.3%) women reported being married, while 82 women (17.05%) had only attained primary school-level education. Notably, women with tertiary education accounted for only 1.6% of the records reviewed.

Despite KNH being located in the capital city, Nairobi, 263 patients (54.68%) reported residing in areas that were up to 3 hours from Nairobi by road. For women with documented occupations, 119 (24.7%) were reported as being self-employed, and 117 (24.3%) were housewives. Parity ranged from 0 to 13, with a mean and median number of 5 children. Over half (n=264, 54.9%) of the women reported having 5 or more children.

Of the 225 women of known HIV status, 164 (34.1%) were HIV-negative. Only 43 (8.9%) of the women reported a history of Pap smear testing, while 12 (2.5%) reported never having undergone cervical cancer screening.

### Clinical Presentation

The most reported histological types of cervical cancer were squamous cell carcinoma (n=406) and adenocarcinoma (n=30).

In 2008, KNH was using the pre-2009 International Federation of Gynaecology and Obstetrics' classification system to stage cervical tumours.^13^ Most women (n=406) were diagnosed at advanced stages – predominantly stages 2B (n=131), 3A (n=55), and 3B (n=140).

Comorbidity with other NCDs was considered, but analyses were restricted to diabetes, heart disease, and hypertension. Of the total study population (N=481), 5 (1.0%) women had diabetes, and 26 (5.4%) had hypertension, while none suffered from heart disease. Out of the 28 women with NCD comorbidity, 3 suffered from both diabetes and hypertension. The prevalence of NCD comorbidity was low at 5.8% in this study population.

### Treatment Options

The full spectrum of cancer treatment available at KNH was surgery (both radical and total abdominal hysterectomy), radiotherapy, and adjuvant chemotherapy (mainly with cisplatin and fluorouracil). We confirmed that out of the 481 women, 66 (13.7%) underwent surgery as part of their treatment plan. A total of 298 (62.0%) women received radiotherapy, with (n=36) or without (n=263) surgery; and, of these, 185 (62.1%) women completed treatment.

In contrast, 66 (13.7%) women received both chemo-therapy and radiotherapy, either with (n=9) or without (n=57) surgery. Only 40 (60.6%) women completed the chemoradiation treatment plan. Women who either underwent surgery only (n=12) or had surgery and chemotherapy (n=9) were grouped together as “other” treatment and represented only 4.4% of the study population. Of the 21 “other treatment” women, 20 (95.2%) completed treatment.

A fifth (n=96, 20.0%) of the medical records provided no evidence of any treatment received.

### Bivariate Analysis

A total of 396 (82.3%) patients were lost to follow-up. There were 50 deaths (10.4%) and only 35 women (7.3%) were reported to be alive 5 years after the initiation of treatment. Based on bivariate analysis, none of the sociodemographic variables directly influenced patient outcomes at KNH, as all P values were >.05 (**Table 1**).

**TABLE 1.** Cross-tabulation of Patient Outcomes vs Sociodemographic and Selected Characteristics

Variables	Cases^a^ n	Deaths n (%)	LTFU n (%)	Alive at 5 Years n (%)	X^2^ *P* Value
Overall	481	50 (10.4)	396 (82.3)	35 (7.3)	
Age, years					
20–39	115	14 (12.2)	96 (83.5)	5 (4.4)	.83
40–49	147	13 (8.8)	123 (83.7)	11 (7.5)	
50–59	111	12 (10.8)	89 (80.2)	10 (9.0)	
60+	108	11 (10.2)	88 (81.5)	9 (8.3)	
Marital status					
Never married/Single	35	6 (17.1)	29 (82.9)	0 (0.0)	.36
Married	194	27 (13.9)	155 (79.9)	12 (6.2)	
Divorced/Widowed	76	15 (19.7)	55 (72.4)	6 (7.9)	
Education					
None	29	12 (41.4)	17 (58.6)	0 (0.0)	.16
Primary	82	26 (31.7)	55 (67.1)	1 (1.2)	
Secondary	37	6 (16.2)	29 (78.4)	2 (5.4)	
Tertiary	8	1 (12.5)	7 (87.5)	0 (0.0)	
Access to care					
Residing in Nairobi	71	13 (18.3)	52 (73.2)	6 (8.5)	.05^b^
≤3 hours from Nairobi by road	263	27 (10.3)	214 (81.4)	22 (8.4)	
>3 hours from Nairobi by road	141	9 (6.4)	125 (88.7)	7 (5.0)	
Occupation					
Casual/Retired/Unemployed	52	10 (19.2)	42 (80.8)	0 (0.0)	.20
Housewife	117	16 (13.7)	94 (80.3)	7 (6.0)	
Professional	18	0 (0.0)	17 (94.4)	1 (5.6)	
Self-employed	119	21 (17.7)	89 (74.8)	9 (7.6)	
Parity					
0	4	1 (25.0)	2 (50.0)	1 (25.0)	.39
1	21	2 (9.5)	19 (90.5)	0 (0.0)	
2–4	165	15 (9.1)	141 (85.4)	9 (5.5)	
5+	264	28 (10.6)	215 (81.4)	21 (8.0)	
HIV status					
Positive	61	10 (16.4)	49 (80.3)	2 (3.3)	.22
Negative	164	20 (12.2)	131 (78.9)	13 (7.9)	
Pap smear screening					
Yes	43	3 (7.0)	36 (83.7)	4 (9.3)	.14
No	12	3 (25.0)	9 (75.0)	0 (0.0)	

a The number of cases for each characteristic is variable across different categories, because data recording in medical records varied, and analysis was confined to the data available.

b Borderline statistical significance relative to a *P*=.05 significance level.

Abbreviations: X^2^, chi-square test; LTFU, lost to follow-up.

For clinical variables, no statistically significant associations between either histological type (X^2^=1.1, 4 degrees of freedom [df]; *P*=.90) or NCD comorbidity (X^2^=7.7, 6 df; *P*=.26) and patient outcomes were found. However, strong statistical associations of *P*<.001 were detected between stage of disease (X^2^=30.1, 6 df), treatment received (X^2^=52.7, 6 df), and treatment status (ie, whether or not treatment was initiated and completed) (X^2^=53.4, 4 df), and patient outcomes.

### Kaplan–Meier Survival Analysis

Five variables – age, access to care, stage of disease, treatment received, and treatment status – were included in the Kaplan–Meier survival analysis. Following the application of the logrank test, age remained statistically non-significant (*P*=.70). Access to care was statistically significant (*P*=.012) while stage of disease, treatment received, and treatment status were all highly significant (*P*<.001).

The Kaplan–Meier survival curves for stage of disease and treatment received are provided in the **Figure**.

**FIGURE:**
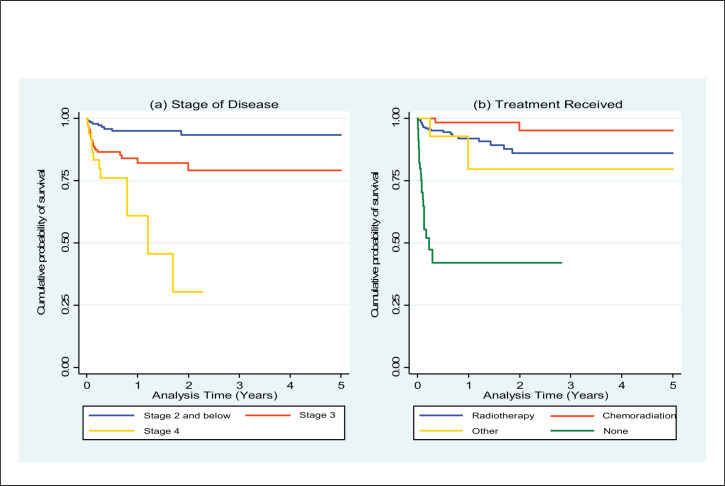
Cumulative Probability of Survival in Women with Cervical Cancer, by Stage of Disease and Treatment Received (N=481)

### Mortality Hazard Ratio Analyses

There were 50 known deaths among women followed up for a total of 494 person years. The average period of follow-up was slightly over 1 year – approximately 13 months – ranging from a minimum follow-up of 1 day to a maximum of 5 years. The overall 5-year survival was estimated as 59.0%.

For age, we observed increments in multivariate hazard ratios across the age groups compared to univariate values. The P values associated with the multivariate hazard ratios were all less than .05; therefore, observed differences in survival were not statistically significant. However, from the multivariate hazard ratios, the 60 years and above age group was at the greatest risk of dying.

Based on multivariate hazard ratios, deaths among patients residing outside of Nairobi appeared less likely to be reported to KNH. Both univariate and multivariate hazard ratios for stage of disease consistently demonstrate that the risk of dying increased with disease stage.

Concerning treatment received, women who received chemoradiation had the lowest risk of dying, although this did not achieve statistical significance. Women who either had surgery or surgery with chemotherapy – the “other” group – were more than 8 times more likely to die compared with those who had radiotherapy as part of their treatment plan. Also, women who completed their recommended treatment plans were least likely to die while those who never started treatment were at the greatest risk of dying.

Analyses suggest that the major determinants of survival among patients with cervical cancer were stage of disease at diagnosis, type of treatment given to the patient, and whether or not a patient initiates and completes treatment (**Table 2**).

**TABLE 2.** Univariate and Multivariate Estimates Using Cox Regression of Mortality Hazard Ratios and 95% Confidence Intervals

	Univariate				Multivariate		
		95% CI				95% CI	
Factors	Hazard Ratio	Lower Limit	Lower Limit	*P* Value^a^	Hazard Ratio^b^	Lower Limit	Upper Limit
Age, years							
20–39	1.00	—	—	—	—	—	—
40–49	0.64	0.30	1.36	.36	0.68	0.29	1.55
50–59	0.76	0.34	1.67	.61	1.25	0.53	2.93
60+	0.78	0.35	1.72	.40	1.46	0.61	3.50
Access to care							
Residing in Nairobi	1.00	—	—	—	—	—	—
≤3 hours from Nairobi by road	0.53	0.27	1.03	.15	0.58	0.27	1.22
>3 hours from Nairobi by road	0.28	0.12	0.68	.04	0.39	0.15	0.97
Stage of disease							
Stage 2 or below	1.00	—	—	—	—	—	—
Stage 3	3.74	1.75	8.00	.01	3.12	1.37	7.07
Stage 4	8.22	3.42	19.76	<.001	5.50	2.18	13.89
Treatment received							
Radiotherapy	1.00	—	—	—	—	—	—
Chemoradiation	0.29	0.07	1.24	.21	0.39	0.09	1.72
Other	1.50	0.35	6.38	.01	8.89	0.61	49.04
None	13.09	7.07	24.25	.70	1.44	0.22	9.52
Treatment status							
Completed	1.00	—	—	—	—	—	—
Incomplete	5.11	2.12	12.31	<.001	7.60	2.79	20.66
Never started	42.26	17.71	100.81	<.001	28.25	3.75	212.94

a *P* values for multivariate hazard ratios;

b Hazard ratios adjusted for age, access to care, stage of disease, treatment status, and treatment received.

Abbreviation: CI, confidence interval.

## DISCUSSION

### Sociodemographic and Selected Patient Factors

None of the sociodemographic factors considered showed a significant association with patient outcomes; however, evidence suggests most are associated with late-stage diagnosis and patient outcomes. Older age, in particular, has been linked to late-stage diagnosis^14,15^ and poor survival outcomes^16–18^ and has been shown to be a factor in treatment defaults leading to poor survival outcomes.^19^

Low education levels have been linked to poor uptake of screening services, increased cervical cancer incidence, and late-stage diagnosis.^14,20–24^ In previous studies, poor education has been closely associated with low socioeconomic status and residence in a medically underserved area. Understandably, a woman's residence in a medically under-served area has also been found to contribute to late-stage diagnosis resulting in poor patient outcomes.^25,26^ In 2008, KNH was the only health facility in the country offering radiotherapy and hosting a comprehensive cancer treatment centre in the country. This explains why a majority of patients reported residing outside Nairobi and travelling long distances to seek care.

Socioeconomic status likely influenced patient outcomes in this study as reported in other studies.^14,25,27^ In Kenya, similar to other LMICs, patients incur significant out-of-pocket expenditure to access medical services^27–29^ due to low per capita expenditures on health by governments.^30^ Despite financial inability being the main reason behind delayed, prolonged, or interrupted treatment, no statistically significant association could be detected between occupation and patient outcomes. It is, therefore, likely that occupation, as recorded in patient files and categorised for our analysis, was not a good measure of socioeconomic status.

In India, having many children at home created a burden to cervical cancer patients resulting in treatment defaults.^19^ This shows that high parity can indirectly contribute to poor patient outcomes in LMICs through increased competition for limited household resources. However, this was not reflected in our study.

Cervical cancer has been classified as an AIDS-defining illness in women with HIV infection, which is a recognised prognostic indicator of poor treatment outcomes for cervical cancer.^31^ Our study findings appear to contradict previous studies whereby HIV-positive cervical cancer patients were more likely to be diagnosed at a later stage, have poorer responses to treatment, exhibit higher rates of recurrence and undergo rapid disease progression compared to HIV-negative women.^31–33^

For this study population, few women had their screening history documented, which contributed to difficulties in accurately establishing the statistical significance of the relationship between screening history and patient outcomes. It would have been expected that regular or previous screening would be associated with positive patient outcomes resulting from early diagnosis.

From the above, it is evident that in low-resource settings, such as Kenya, 2 key factors specifically limit the effectiveness of retrospective cancer survival studies. First, inconsistent documentation of patient histories, sociodemo-graphic, and contact information within primarily manual and paper-based systems. Second, the lack of centralised vital registration and national health sector systems makes it difficult to follow up with patients once they leave a specific health facility. As a result, a number of statistically significant relationships were likely undetected during data analysis.

### Clinical Factors

Most women were diagnosed at advanced stages of disease. This is similar to several studies that have collectively established that more than 80% to 90% of women across sub-Saharan Africa present with late-stage cervical cancer.^24,27,34^

In their 2002 systematic review, Grossman et al^35^ found an association between hypertension and increased mortality among cancer patients. Other studies that have shown that type 2 diabetes resulted in poor oncological outcomes in patients with early stage cervical cancer.^36,37^ These proposed associations were not reflected in this study, which could be the result of both low overall prevalence of NCD comorbidity in the study population and a small number of patients undergoing or completing chemoradiation.

With regards to treatment options, chemoradiation with cisplatin is the accepted standard treatment for locally advanced disease.^38^ Despite this, the critical component of treatment plans for the women in this study was radiotherapy, with or without surgery, depending on disease stage.

The strong statistical associations between patient outcomes versus stage of disease, treatment received, and treatment status at KNH suggests that while the loss to follow-up was exceedingly high, the few deaths reported followed a distinct pattern allowing for these relationships to be detected. Furthermore, compared to patient history and socio-demographic information, clinical data were relatively well documented, as they are the primary focus of medical records.

### Overall Survival

The overall survival rate of 59% (12 deaths per 100 person years) in our study population was likely an underestimation owing to the high proportion of women lost to follow-up. As almost all reported deaths occurred within the first year of follow-up, the actual survival rate over 5 years is likely to be less than 25%, as has been observed in the Gambia and Uganda.^10^ This suggests that a review of medical records can yield more accurate survival estimates for a 1-year period or less, but inaccuracies will increase as the period of follow-up increases if additional measures to ascertain patients' vital status are not taken. Notwithstanding the study's limitations, the results suggest that stage of disease at diagnosis, treatment received, and whether or not treatment was completed were major predictors of survival among women treated for cervical cancer in Kenya.

Patients diagnosed with stage 4 cervical cancer demonstrated the greatest risk of dying. Globally, stage 4 cervical cancer has been shown to have a poor prognosis and an extremely low survival rate of 15% to 16%.^39^ In many LMICs, late-stage diagnosis coupled with incomplete treatment for advanced cancer contributes to mortality, as 80% of patients already have incurable disease when first diagnosed.^40^ Maranga et al^26^ additionally raises concerns over the possibility of “under-staging” – wherein women had more advanced disease than was diagnosed – impacting negatively on patient outcomes.^27^ Similar to their study at KNH, we noted during our study that initial staging and tumor response were primarily assessed using digital vaginal examination because few patients could afford imaging tests, such as ultrasound, x-ray, computed tomography, or magnetic resonance imaging. Consistency of these results with international studies is yet another reminder of the importance of scaling up cervical cancer screening and diagnostics in LMICs.^41^

There was no statistically significant difference in the risk of dying between patients receiving chemoradiation compared with those receiving radiotherapy. Chemoradiation has been demonstrated to increase the chances of survival among cervical cancer patients.^42,43^ One hypothesis to explain this phenomenon is the prohibitive cost of treatment.^40,44^ While the cost of chemotherapy plus radiotherapy is significantly higher than other treatments, it is plausible that many patients recommended for either radiotherapy or chemoradiation are equally unlikely to either start or complete treatment due to limited finances. Furthermore, a significant number of women were rendered ineligible for chemotherapy owing to complications from advanced disease (severe anemia or hydronephrosis) or advanced age (60 years and above). These reasons may explain why the difference in risk of dying was not statistically significant between the 2 groups despite expectations that patients recommended for chemoradiation would have reduced risk.

From reviewing the records, we noted that the period from diagnosis to commencement of treatment took an average of 2 to 3 months for most patients. This phenomenon was also reported by Maranga et al,^26^ citing the main reasons for delay as financial constraints, difficulties with travelling, inability to gain admission to crowded hospital oncology wards, and queues of patients awaiting treatment with the single radiotherapy machine at KNH.^27^ To that end, it is evident that organisational delays in accessing diagnostic and treatment services additionally contribute to poor patient outcomes.

Since 2008, multiple initiatives aimed at improving cancer management in Kenya have been launched. The Ministry of Health – in partnership with reproductive health partners – are rapidly expanding access to visual inspection screening methods and ensuring that basic treatment with cryotherapy for precancerous lesions is widely accessible. The hospital has acquired new radiotherapy machines and, in 2017, officially launched its cancer treatment centre. In January 2016, Kenya's national hospital insurance fund launched revised benefit packages that have enabled more patients to access cancer care. Moreover, between 2010 and 2012, 4 comprehensive private health sector cancer treatment centres have been established. Cancer survival studies are urgently needed to examine the impact of these strategies on access to care and overall survival.

### Ascertaining Patients' Vital Status in Future Studies

Four methods of study participant follow-up are recommended: provision of incentives, use of mailing addresses, telephone follow-ups, and home visits.^45^ Owing to the lack of resources available to existing oncology programmes in sub-Saharan Africa, incentives, use of mailing addresses, and home visits may not be practical. However, the use of mobile phones in Africa has grown exponentially in the last decade and may provide an effective way for conducting active surveillance of cancer patients.^46^ Follow-up by telephone could go beyond ascertaining the patients' vital status by providing advice on medications, clarification of missing or unclear information from medical records, psychosocial support through training callers to handle difficult topics – such as coming to terms with medical illness – and referrals to any organisations and foundations offering supportive services to cancer patients and their families.

Additionally, there needs to be a greater emphasis on prospective cancer survival studies, because the rapport and trust built during recruitment of study participants would facilitate long-term follow-up in a context where cancer patients exhibit high mobility by seeking care in multiple facilities.

Additionally, cancer survival studies should be conducted within a national cancer research network, which would harmonise data collection tools and set up a vital registration database for cancer patients. Using data-sharing agreements, this database could then be used for subsequent studies and help improve data quality.

### Limitations

Patient medical records may have been missed for various reasons: reviews of deceased patient files at the RTC were not done, patients died on arrival to the hospital before diagnostic confirmation, and the facilities may have misplaced files. Furthermore, the women who registered for treatment at KNH were a select subset of women with cervical cancer, as more seriously ill women and women with fewer resources may not have had access to KNH.

Lack of a centralised national database for vital registration prevented determination of how many patients may have died outside KNH. Also, lack of a centralised national health system database prevented effective follow-up of patients who may be continuing care in alternative health facilities across the country.

## CONCLUSION

Late-stage diagnosis, treatment defaults, and constrained oncological health services undoubtedly contribute to the high mortality rates from cervical cancer in Kenya. Reviewing medical records is an integral component of cancer survival studies that needs to be coupled with innovative strategies to ascertain patients' vital status in regions where vital registration systems are limited in coverage, and national health system databases are either nonexistent or limited in scope.
